# High Performance of PEDOT:PSS/n-Si Solar Cells Based on Textured Surface with AgNWs Electrodes

**DOI:** 10.1186/s11671-018-2462-0

**Published:** 2018-02-14

**Authors:** Xiangyu Jiang, Pengbo Zhang, Juan Zhang, Jilei Wang, Gaofei Li, Xiaohong Fang, Liyou Yang, Xiaoyuan Chen

**Affiliations:** 1 Thin Film Optoelectronic Technology Center, Shanghai Advanced Research Institute, Chinese Academy of Sciences, Shanghai, 201210 China; 2 University of Chinese Academy of Sciences, Beijing, 100049 China; 3 School of Physical Science and Technology, Shanghai Tech University, Shanghai, 201210 China; 4 Jinneng PV Technology LTD, Jinzhong, 030600 China

**Keywords:** Silver nanowires, PEDOT:PSS, N-Si solar cells, Drop-casting

## Abstract

Hybrid heterojunction solar cells (HHSCs) have gained extensive research and attention due to simple device structure and low-cost technological processes. Here, HHSCs are presented based on a highly transparent conductive polymer poly(3,4ethylenedioxythiophene):poly(styrenesulfonate)(PEDOT:PSS) directly spin-coated on an n-type crystalline silicon with microscale surface textures, which are prepared by traditional chemical etching. We have studied interface properties between PEDOT:PSS and textured n-Si by varying coating conditions. Final power conversion efficiency (PCE) could arrive at 8.54% by these simple solution-based fabrication processes. The high conversion efficiency is attributed to the fully conformal contact between PEDOT:PSS film and textured silicon. Furthermore, the reflectance of the PEDOT:PSS layer on textured surface is analyzed by changing film thickness. In order to improve the performance of the device, silver nanowires were employed as electrodes because of its better optical transmittance and electrical conductivity. The highest PCE of 11.07% was achieved which displayed a 29.6% enhancement compared with traditional silver electrodes. These findings imply that the combination of PEDOT:PSS film and silver nanowire transparent electrodes pave a promising way for realizing high-efficiency and low-cost solar cells.

## Background

Approximately 90% of global photovoltaic market is occupied by crystalline silicon solar cells for performing well on both cost and efficiency [1–4]. Using n-crystalline silicon and poly(3,4ethylenedioxythiophene):poly(stylenesulfonate)(PEDOT:PSS) manufactured hybrid heterojunction solar cells (HHSCs) are favored by researchers [5]. The properties of dopant-free, vacuum-free, low-temperature, and solution-proceeded fabrication procedures determine that PEDOT:PSS/n-Si heterojunction solar cells have a series of superiorities on the cost [6, 7]. The highest reported power conversion efficiency (PCE) of HHSCs is 16.2% created by Jian He et al. [8]. The efficiency gap between HHSCs and conventional silicon cells is gradually narrowing.

In HHSCs, crystalline silicon, having high mobility and long minority carrier lifetime, is an active absorber for collecting photons to produce photo-generated carriers and transporting electrons. On the other hand, the PEDOT:PSS layer, with high transmittance(85% for 100 nm thickness) and high conductivity(1000 S/cm for Clevios PH1000) [9], works as a transparent conducting hole-transporting layer and optical window [10]. Therefore, the HHSCs have potentials to achieve higher PCE. However, the PCE of HHSCs is greatly restricted to inferior junction quality at the PEDOT:PSS/n-Si interface.

Interface engineering is essential for the PEDOT:PSS/n-Si solar cells because it optimizes the carriers transmission and separation and reduces the interface recombination velocity [11]. Several common methods are used to improve the PCE of PEDOT:PSS/n-Si heterojunction solar cells: reducing the thickness of crystalline silicon by depositing film crystalline silicon, applying colloidal quantum dot, texturing silicon surface into nanostructures, introducing the back surface field(BSF), and applying silicon nitride or silicon oxide as a passivation layer [5, 6, 12–21]. However, the contact properties of PEDOT:PSS with textured substrate has seldom been considered, which raises the *J*_sc_ and efficiency of PEDOT:PSS/n-Si hybrid solar cells from the perspective of interface engineering.

Our works are carried on the Si surface textured by traditional alkaline solution process [22]. The uniformity of PEDOT:PSS film thickness is more difficult on textured Si than that on the plane ones. Unlike traditional electrodes, the silver nanowires (AgNWs) electrodes have superiority on optical transmittance. To our knowledge, the diluent of silver nanowires were difficult to coat on textured polymer film. The coating methods such as rod-coating or spin-coating cause the presence of nonuniformity and damage. In this paper, PEDOT:PSS/n-Si solar cells were fabricated with silver nanowire electrodes by means of drop-casting. The novel electrodes application on the cells provides a feasible, low-cost, and high-efficiency metallizing process.

## Methods

### Preparation of Textured Si Substrates for HHSCs

N-Si(100) Czochralski (CZ) wafers (thickness 210 μm, 1–3 Ω cm) were used as substrates. Samples were cleaned using standard cleaning solution (SC1 and SC2) and then polished in a high concentration of KOH solution at 75 °C for 2–3 min to remove the damaged layer. After standard cleaning process, the substrates were textured into a double-sided random pyramids structure by immersing in the mixed solution of KOH (2 wt.%) and isopropanol (2 wt.%) at 75 °C for 15–20 min. The height of random pyramids on textured silicon surface is about 1 μm. Followed with another RCA cleaning process, the textured samples were immersed in diluted HF solution for 0.5–1 min to obtain clean oxide-free silicon surfaces.

### Fabrication of Si/PEDOT:PSS Hybrid Solar Cells

**Fig. 1:**
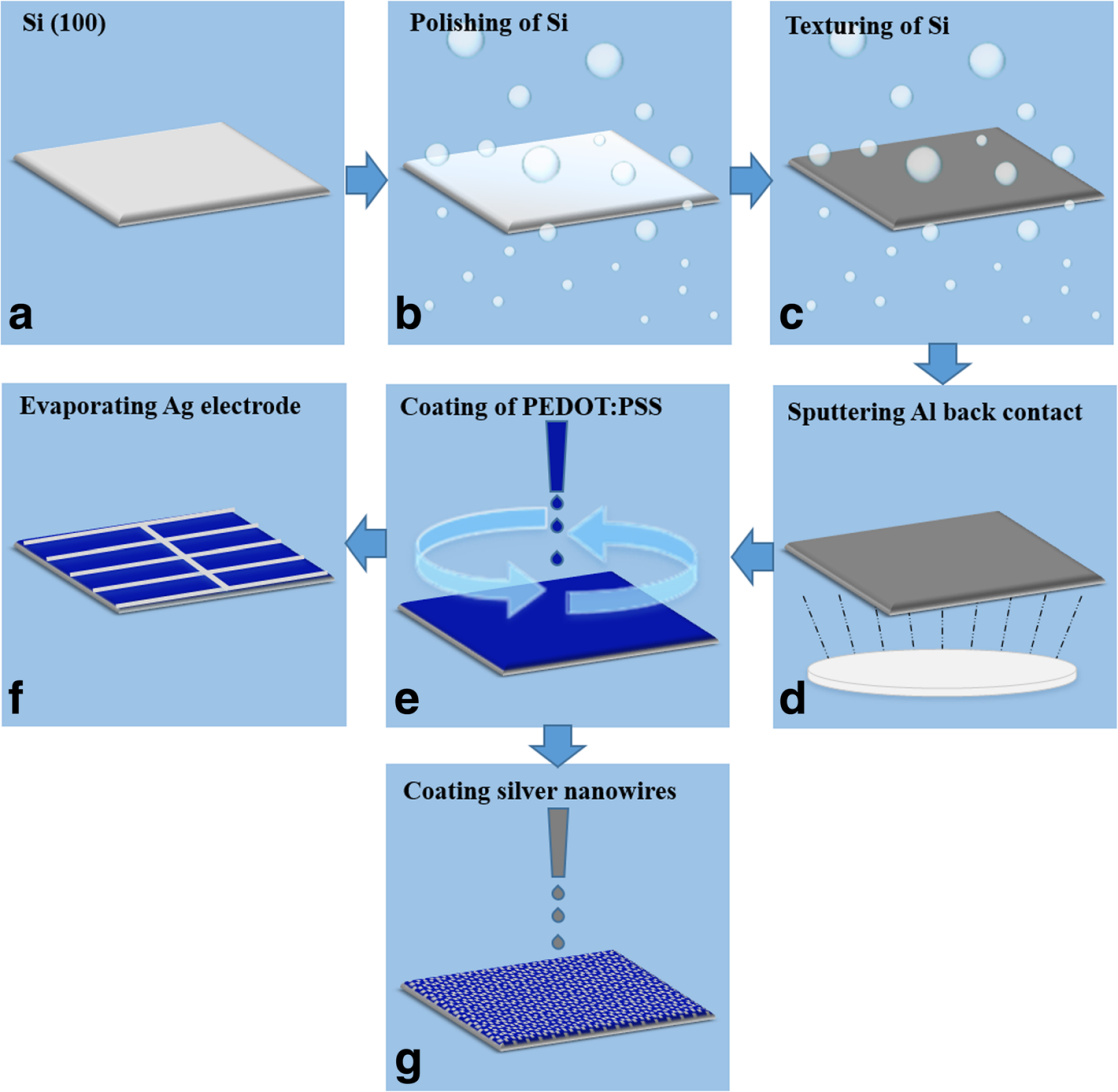
Schematic of preparing the n-Si/PEDOT:PSS solar cells with (**a-f**) Ag grid electrodes or (**a-e, g**) silver nanowires electrodes

Schematic diagrams of the technological process were displayed in Fig. 1. The aluminum back contact (200 nm) was prepared on the back surface of the samples using magnetron sputtering. The dimethyl sulfoxide (5 wt.%, DMSO) and fluoride surfactant (0.1 wt.%, Capstone FS31) were distributed into the PEDOT:PSS (Clevios PH1000) solution to improve the electrical conductivity and coating quality. The mixed PEDOT:PSS solution was spin-coated on the top of the wafer at different coating rates. Then, the samples were annealed in an oven at 130 °C for 15 min to remove the solvents to form a highly conductive p-type organic thin film. Silver grid electrodes (200 nm) were thermally evaporated on the top surface of devices through a shadow mask. In addition, the alternative silver nanowire electrodes were prepared on the top of samples by drop-casting silver nanowires dispersion. The silver nanowires were dispersed in isopropyl alcohol (5 mg/ml, 50 nm in diameter and 100–200 μm in length, XFNANO). Subsequently, samples were dried in an oven at 150 °C for 5 min to remove the solvents.

### Device Characterization

The reflectance spectra measurements were carried out with an integrating sphere. The scanning electron microscope (SEM) photos were obtained using S4800 Hitachi. The *J−V* characteristics of the cells were performed by an Oriel solar simulator (94063A, Newport Corporation), 450 W Xe lamp, simulated air mass AM 1.5 solar spectrum irradiation source at 100 mW/cm^2^, mono-crystalline reference cell, and Keithley 2400 source meter. The absorption spectral lines were measured using an ultraviolet spectrophotometer (UV-8000 s Shanghai Precision instruments Co. Ltd). The transmittance measurements of the PEDOT:PSS film were gained by QEX10 (PV Measurements, Inc.). The square resistance was carried out by employing a four-probe sheet resistance tester (SDY-4, Guangzhou Semiconductor Materials Research Institute).

## Results and Discussion

**Fig. 2:**
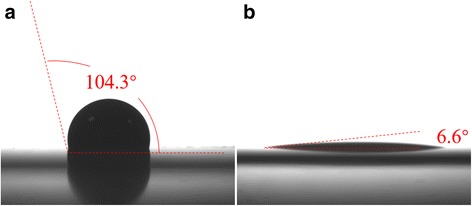
The contact angle between wafer and PEDOT:PSS solution (**a**) without FS31 and (**b**) with FS31

**Fig. 3:**
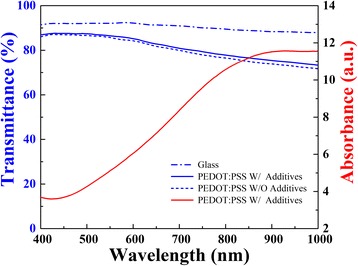
The red line is the absorbance spectrum of PEDOT:PSS with additives(DMSO and FS31) at wavelength from 400 to 1000 nm. The blue lines are the transmittance spectra of PEDOT:PSS film with and without additives and reference glass at wavelength from 400 to 1000 nm, respectively

Improving optical and electrical properties by applying additives into PEDOT:PSS film would enhance the performance of solar cells. A “secondary doping” method is used to enhance the conductivity of the organic layer by adding dimethylsulfoxide (DMSO) to PEDOT:PSS compound [23]. The electrical conductivity of PEDOT:PSS solution can be greatly increased by adding extra DMSO of 5 wt.% [10, 23, 24]. The sheet resistance of PEDOT:PSS layer spin-coated on glasses was 136 Ω/□ at 2000 rpm. However, we found the contact angle between hydrophobic silicon surface and PEDOT:PSS solution was 104.3° (Fig. 2a), which extremely obstructed the spin-coating quality. A useful method is mixing fluoride surfactant into PEDOT:PSS solution to reduce the contact angle [25]. Figure 2 shows the differences of contact angles between wafer and PEDOT:PSS solution (with and without FS31 of 0.1 wt.%). As a result, the contact angle of the PEDOT:PSS solution on the hydrophobic silicon surface was found remarkably decreased. The optical transmittance of PEDOT:PSS film with and without additives coated at 5000 rpm on glass are showed in Fig. 3. The PEDOT:PSS film demonstrates an optical transmittance of 85% contrast with the reference glass. With the applying of DMSO and FS31, the transmittance of PEDOT:PSS could be increased slightly at wavelength from 600 to 1000 nm. The spectra exhibit higher optical characteristics between 400 and 1000 nm, which makes it optimal as an optical window in PEDOT:PSS/n-Si solar cells. Besides, the uniformity of film thickness has been improved in the spin-coating process. In general, the additives enhance the optical properties of PEDOT:PSS and the contact performance between the textured silicon surface and PEDOT:PSS layer.

**Fig. 4:**
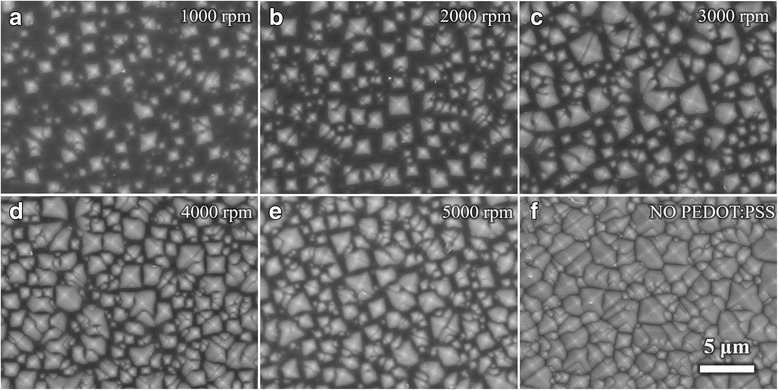
The SEM top view images of textured Si with PEDOT:PSS layer. **a**–**e** coating rates range from 1000 to 5000 rpm, and **f** has no PEDOT:PSS layer. The scale bars in **a**–**f** are the same

**Fig. 5:**
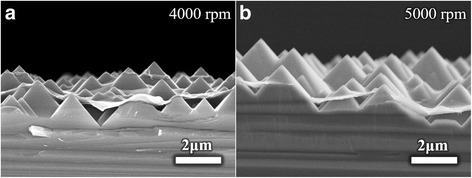
The cross-sectional view of textured Si coated PEDOT:PSS film (**a**) with 4000 rpm and (**b**) with 5000 rpm

Traditional industrialized texturing process is adopted to form light trapping structure. Due to the anisotropic reaction rates of silicon wafer in a hot alkaline solution, the front and back surfaces of silicon are etched into micro-pyramidal structure with random sizes. The corresponding pyramidal surface SEM image is illustrated in Fig. 4f. The complex structure on the silicon sets barriers to achieve uniform PEDOT:PSS film and fabrication processes. To overcome the thickness uniformity problem on the textured silicon surface, spin-coating has advantages over other coating methods. Figure 4a–e depicts top views of PEDOT:PSS film on the pyramid structure fabricated at spin-coating rates from 1000 to 5000 rpm and 8000 rpm, respectively. Figure 5 shows the cross-sectional views of substrate-coated PEDOT:PSS at **a** 4000 rpm and **b** 5000 rpm. At a low rate, the surface tension of the PEDOT:PSS solution makes it hard to penetrate into the valleys surrounded by pyramids. The increasing spin-coating rate could enhance the penetration rate and the adhesiveness of PEDOT:PSS solution on the micro-pyramidal surface [26]. The coverage area is expanded with spin-coating rate; the voids become so small that the PEDOT:PSS could nearly conformal contact with the textured substrates. As a result, the air voids under the PEDOT:PSS film as showed in Fig. 5 gradually become smaller [27]. Besides, the contact area and contact quality between the textured structure and PEDOT:PSS film are gradually improved as spin-coating rate increases. As coating rates increase, the thickness of PEDOT:PSS film reduces, the pyramids gradually emerge from PEDOT:PSS film, and the flatness of substrate decreases correspondingly.

**Fig. 6:**
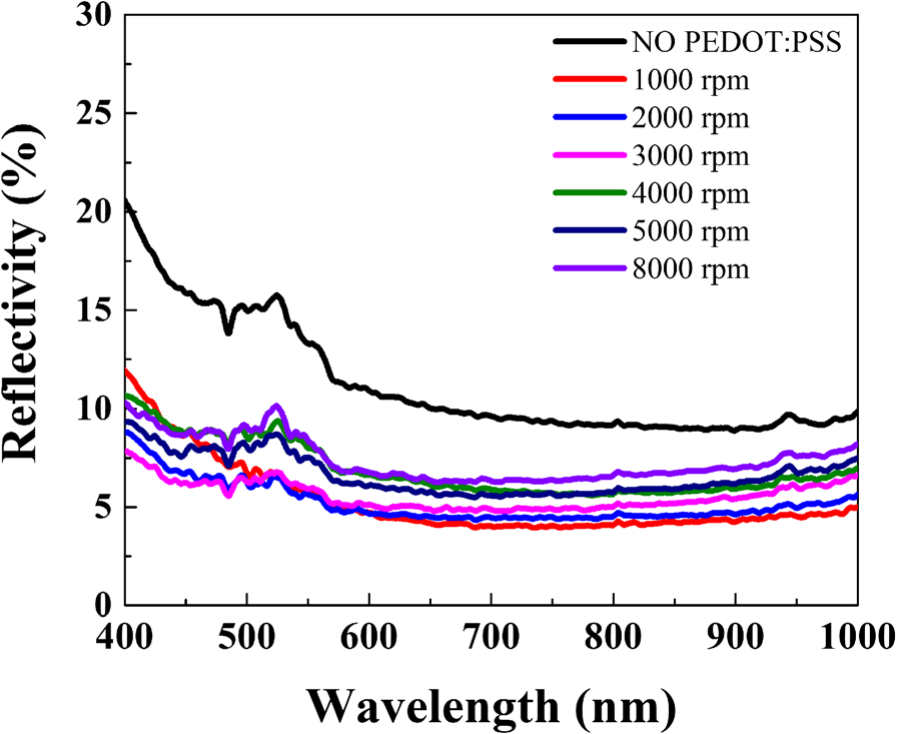
The reflectance curves of textured Si coated with PEDOT:PSS layer at different coating rates from 1000 to 5000 rpm, 8000 rpm, and no PEDOT:PSS

However, the coating conditions strongly affected the morphologies of devices. To characterize the optical properties of the substrates, reflection spectra were recorded for samples with various coating conditions of PEDOT:PSS. As exhibited in Fig. 6, the reflectance of the original textured Si substrate is ~ 10 to 20% due to the effective light trapping and light scattering caused by increasing optical path length of incident light between the micro-pyramidal structures on silicon surface. The experimental results clearly demonstrate that the stacking of PEDOT:PSS film on micro-pyramidal structures obviously improves the antireflection of devices by ~ 5%. In the wavelength range of 600 to 1000 nm, the reflectance appears to be dependent on the coating rates. However, the reflectance seems to be irregular in short wave band. Especially for the sample at 1000 rpm, the reflectance seems to be higher than that under other rates. Considering the relationships between thickness of PEDOT:PSS film and its light reflectance, Fig. 3 shows the absorption spectrum and the transmittance spectrum of PEDOT:PSS film coated on glass at 5000 rpm at wavelength from 400 to 1000 nm. The absorption of PEDOT:PSS in wavelength from 600 to 1000 nm is relatively larger than that in short wave band, and the reflectance is proportional to the coating rate. However, the absorption coefficient at wavelength from 400 to 600 nm is comparatively lower. Moreover, the flatness of the surface occupies the major factor of effecting the reflectivity. When the film is relatively thick, the pyramids are nearly submerged and the surface flattens out, which determines the reflectance of the PEDOT:PSS film on silicon surface. Based on the above discussion, we tentatively put forward that the reflectance of PEDOT:PSS layer on textured surface is influenced by both the dielectric layer absorption and the surface flatness.

**Fig. 7:**
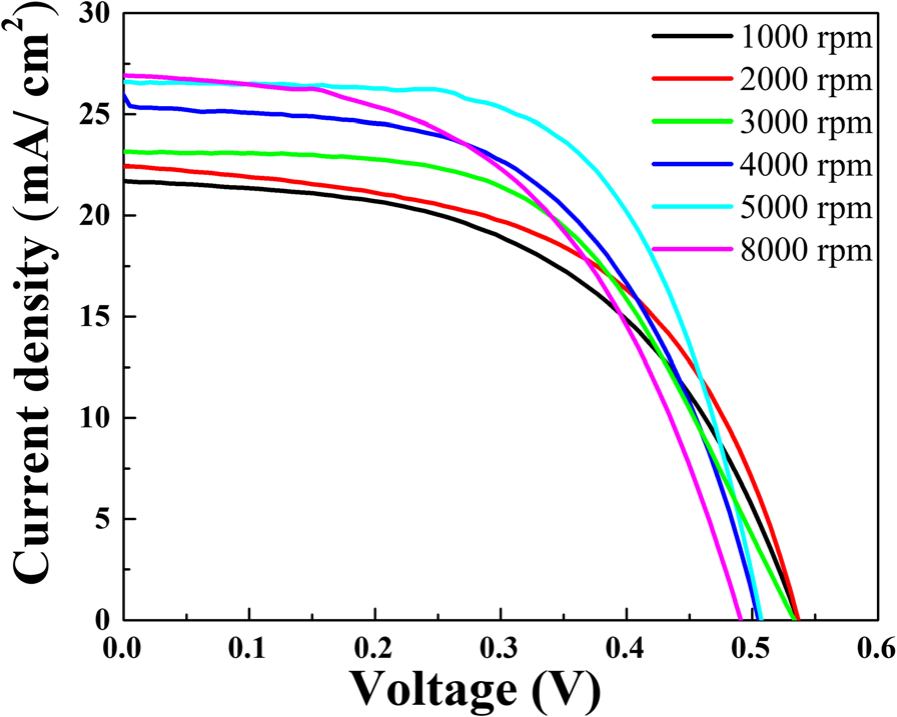
The *J-V* curves of the HHSCs with different PEDOT:PSS coating rates from 1000 to 5000 rpm and 8000 rpm at AM1.5

The role of contact properties and thickness of PEDOT:PSS film for solar cells performance has also been explored. The light current density–voltage (*J–V*) curves for the HHSCs with different PEDOT:PSS coating rates are shown in Fig. 7, and the homologous electrical characteristics are summarized in Table. 1. The device with evaporated silver grid electrodes has a peak conversion efficiency of 8.54%. The total area of the device and the electrodes are 20 × 20 mm and 40 mm^2^, respectively. As exhibited in Table. 1, the *J*_sc_, FF, and PCE of PEDOT:PSS/n-Si hybrid cells are correlated with the coating conditions. As coating rates increase, the contact area, contact quality, and film thickness get optimized; the *J*_sc_ of the solar cell gradually raises from 21.68 to 26.88 mA/cm^2^. At low rate, the PEDOT:PSS thin film could not deposit on the bottom of the valleys between pyramids. As shown in Fig. 5, the contact junction areas between PEDOT:PSS film and the top of pyramids are so small that PEDOT:PSS film cannot collect enough charge, resulting in a poor heterojunction [26, 27]. In addition, due to the wide bandgap of PEDOT:PSS, PEDOT:PSS film could reduce the interface recombination velocities and block electrons from recombination at the front surface of device.

**Table 1:** *J-V* characterization of the textured HHSCs with different PEDOT:PSS coating rates from 1000 to 5000 rpm and 8000 rpm at AM1.5

Spin-coating rate (rpm)	*V*_oc_ (*V*)	*J*_sc_ (mA/cm^2^)	Fill factor (%)	Efficiency (%)
1000	0.53	21.68	52.31	6.08
2000	0.53	22.41	54.78	6.58
3000	0.53	23.14	55.18	6.82
4000	0.50	25.40	55.78	7.15
5000	0.51	26.55	62.13	8.54
8000	0.49	26.88	51.68	6.95

In practical application on textured substrates, PEDOT:PSS film thickness could not be adjusted without considering contact properties. The spin-coating process simultaneously restricts film thickness and contact quality [7]. It is known that a relatively high coating rate is very necessary for efficiency improvements. The enhanced heterojunction areas contribute to the separation of holes and electrons and an increase on *J*_sc_. The high-quality interface contact leads to a falling of interface recombination velocity and a significant current boost [11, 18]. Such a fact can be found from Figs. 4 and 5, that there is no massive conductive organic material stacking over the valleys at 5000 rpm. For the reduction of PEDOT:PSS film thickness, textured silicon surface traps more light [26]. The decreased parasitic absorption loss of the thinner PEDOT:PSS layer leads to an enhancement of photons absorbing of the silicon surface, improving photocurrent and cell efficiency. However, when the spin-coating rate reaches 8000 rpm, the open circuit voltage reduces to 0.49 V because PEDOT:PSS film may be too thin to cover the whole Si surface and the heterojunction probably shortens. A thinner film would cause the direct connection between metal electrodes and top of pyramids. Meanwhile, due to the decreased film thickness, the decreased length of P-N junction has an effect on the device performance [23]. And, the nonuniformity of film thickness at 8000 rpm may be especially important on influencing device efficiency. Therefore, the highest performance of PEDOT:PSS/n-Si solar cells occurs at 5000 rpm.

**Fig. 8:**
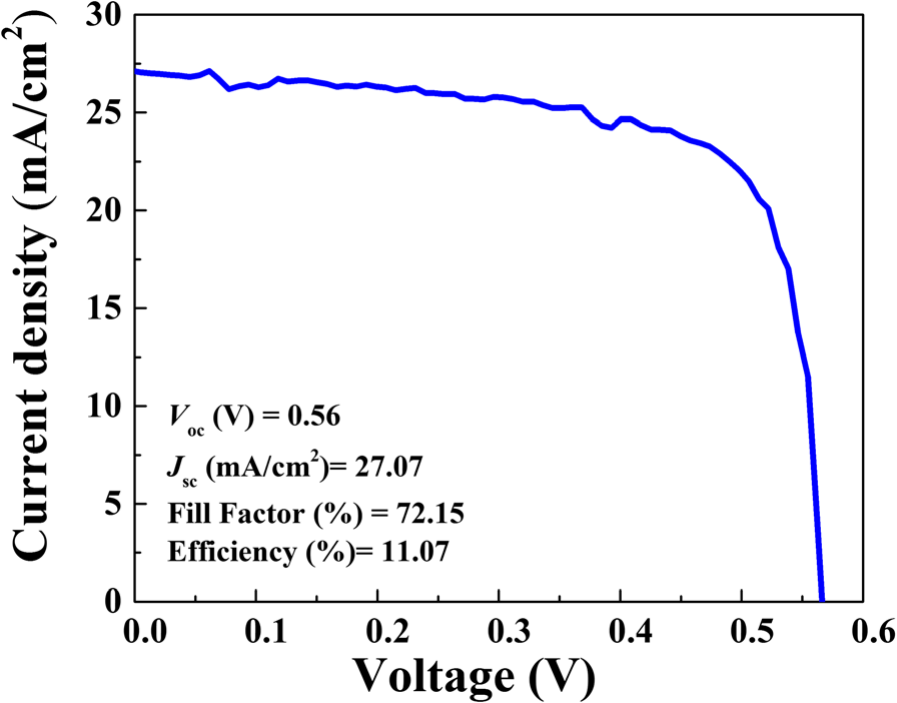
*J*–*V* curves of PEDOT:PSS/n-Si hybrid solar cells with silver nanowire electrodes

**Fig. 9:**
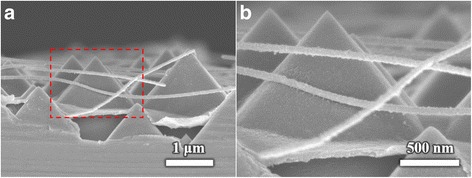
**a** The cross-sectional view of PEDOT:PSS/n-Si solar cells with AgNWs electrodes. **b** The detailed image of red rectangle

The above samples were produced with silver grid electrodes. For using the highly transparent and conductive silver nanowires electrodes, the similar AgNWs film on planer substrates was reported in HHSCs [28, 29]. We have also fabricated devices using AgNWs electrodes with a total area of 20 × 20 mm. When the coating rate of PEDOT:PSS arrived at 4000 rpm, solar cells with silver nanowires electrodes can achieve a highest PCE of 11.07% using drop-casting methods. The measurements are shown in Fig. 8. The SEM image of silver nanowire electrodes on textured substrate is displayed in Fig. 9. The silver nanowires could contact with the pyramids. And, the electrodes contacting area between AgNWs and PEDOT:PSS is larger than that in the devices with silver electrodes. The series resistance of PEDOT:PSS/n-Si solar cells decreases from 0.84 to 0.38 Ω/cm^2^ mainly because the AgNWs film electrodes possess low square resistance of ~ 10 Ω/□. The fill factor and *V*_oc_ could greatly increase from 62.13 to 72.15% and 0.51 to 0.56 V, respectively, because of the reduced series resistance of the devices. Moreover, the plasmonic effect of AgNWs plays a significant role on light-harvesting boost [30–33]. Malika Chalh indicated that the AgNWs (more than 10 μm) can cause excitation of the surface plasmon mode, which could enhance the absorption for a wavelength range between 400 and 700 nm [34]. The surface of the Si substrate is covered with a lot of silver nanowires, which form grids for collecting charge. The enhancement of absorption inside the active layer can be increased, via a coupling between each wire. However, the AgNWs would result in the strong parasitic absorption losses in the metal and active layers. Here, the thicker active layer could reduce the absorption in the AgNWs layer while inducing more absorption in the active layer [35]. Therefore, the device has shown significant enhancement on broadband light absorption employing the plasmonic AgNWs via the efficient scattering of light and plasmonic coupling [36]. With the substitution of AgNWs electrodes, the short circuit current density of the device gets increased from 26.55 to 27.08 mA/cm^2^. It turns out the silver nanowire electrodes are able to achieve higher PCE in the PEDOT:PSS/n-Si solar cells.

## Conclusions

In summary, the mixed PEDOT:PSS solution of DMSO and FS31 achieves higher conductivity and smaller contact angle on the textured hydrophobic surface. The short wavelength reflectivity of PEDOT:PSS layer on the textured surface is influenced by the combined effect of absorption coefficient and flatness of substrate surface. With better contact quality, proper film thickness, and larger contact junction area at optimized coating rate, the performance of the HHSCs gets enhanced. The application of silver nanowire electrodes demonstrated a simple promising fabrication process for getting higher PCE.
